# A Rare Presentation of Cardiac Tamponade from Metastatic Urothelial Carcinoma of the Bladder

**DOI:** 10.1155/2018/6750264

**Published:** 2018-06-19

**Authors:** Sowmya Palam, Ridhima Kapoor, Jacquelyn Kulinski

**Affiliations:** Department of Internal Medicine, Medical College of Wisconsin, 9200 W. Wisconsin Ave., Suite 5100, Milwaukee, WI 53226, USA

## Abstract

A 62-year-old man presented with 2 weeks of progressive dyspnea and chest pain. He was previously diagnosed with high-grade invasive urothelial carcinoma (UC) of the bladder and underwent neoadjuvant chemotherapy followed by radical cystectomy 10 months earlier, resulting in pathologic complete remission. Clinical evaluation and echocardiographic imaging was consistent with a diagnosis of cardiac tamponade. Due to a history of malignancy, the patient was referred for a surgical pericardial window, to include biopsy of the pericardium. Pericardial fluid and pericardial biopsy specimens were consistent with metastatic UC. Cardiac tamponade due to metastatic UC is a rare presentation, and, to our knowledge, there have been only 5 cases reported in the English literature. We report a rare case of cardiac tamponade due to isolated pericardial metastases from high grade UC of the bladder and discuss the symptoms, treatment, and prognosis of this pathologic condition. We also present a brief review of previously published literature. Through this discussion, we would like to emphasize the (1) consideration of cardiac metastases in the differential diagnosis for patients with a history of UC presenting with cardiac or pulmonary symptoms and (2) improved diagnostics with pericardial biopsy and pericardiocentesis over pericardiocentesis alone.

## 1. Introduction

Bladder cancer is the ninth most common malignancy worldwide involving the urinary system. Urothelial carcinoma (UC) is the most prevalent histologic type in the United States and Western Europe and accounts for approximately 90% of bladder cancers [1, 2]. Common sites of metastasis of UC include regional and distant lymph nodes, liver, lung, and bone [3]. However, in rare cases, metastasis to the pericardium can also be seen. Approximately ten percent of autopsied cases of UC have shown cardiac metastasis [4]. However, in most cases, the metastases are clinically silent. We report a rare case of cardiac tamponade caused by metastatic UC of the bladder and discuss the patient's symptoms, management, and prognosis.

## 2. Case Presentation

A 62-year-old man presented with 2 weeks of progressive dyspnea and chest pain. He was previously diagnosed with high-grade invasive urothelial carcinoma (UC) of the bladder. He underwent neoadjuvant chemotherapy followed by radical cystectomy, resulting in complete pathologic remission. On examination, patient was afebrile with a heart rate of 136 bpm, blood pressure of 122/74 mm Hg, respiratory rate of 18 breaths/min, and O_2_ saturation of 98% on room air. Heart sounds were muffled. Heart rhythm was irregularly irregular. Distention of the jugular veins was observed and Kussmaul's sign was present. Although pulsus paradoxus was not observed, other physical findings were extremely concerning for cardiac tamponade. Bibasilar crackles were present on lung auscultation. Laboratory data was remarkable for a creatinine of 1.42 (baseline of 1.08) and international normalized ratio (INR) of 3.6 (on warfarin for a history of pulmonary embolism). His complete blood counts and electrolytes were normal. His electrocardiogram revealed atrial fibrillation with a rapid ventricular rate (RVR) and electrical alternans. Chest radiograph showed enlargement of the cardiac silhouette.

**Figure 1:**
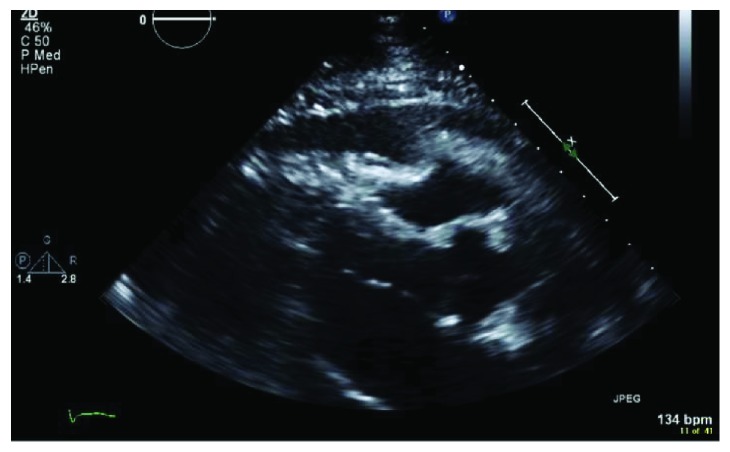
Transthoracic echocardiogram, parasternal long view, demonstrating large pericardial effusion and RV diastolic collapse.

**Figure 2:**
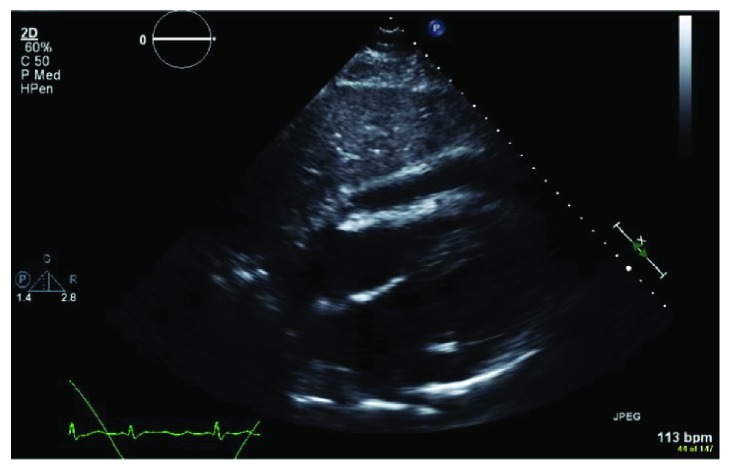
Transthoracic echocardiogram, subcostal view.

**Figure 3:**
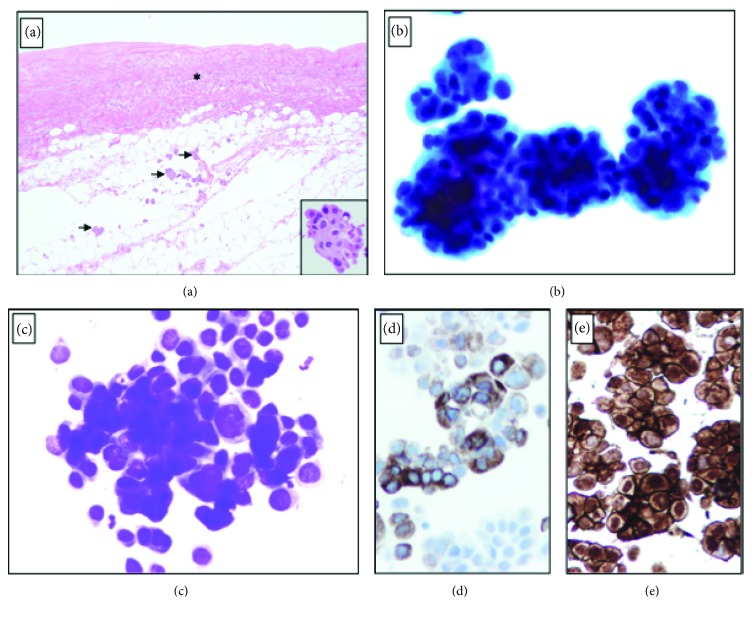
(a) H&E section shows thickening and fibrosis of the pericardium (asterisk) and scattered aggregates of metastatic urothelial carcinoma (black arrows). Insert: high power view of the malignant cells. The pericardial effusion demonstrates 3D clusters of large highly atypical malignant cells (b, Papanicolaou stain), variable in size and hyperchromatic with a high nuclear to cytoplasmic ratio (c, Diff-Quick stain). The malignant cells are immunoreactive for uroplakin and MOC-31 while negative for calretinin (not shown).

Due to high clinical suspicion for cardiac tamponade, a bedside transthoracic echocardiogram (TTE) was obtained which showed a large pericardial effusion with diastolic compression of the right ventricle, suggestive of cardiac tamponade, as well as mild left ventricular systolic dysfunction (see Figures 1 and 2). Patient's INR was reversed to <1.5 and he underwent emergent subxiphoid pericardial window with the removal of 700 ml of turbid, dark, bloody pericardial fluid. He had significant improvement in hemodynamics and respiratory distress. The pericardial fluid cytology and pericardial biopsy were both positive for malignancy, consistent with metastatic UC (see Figure 3).

**Figure 4:**
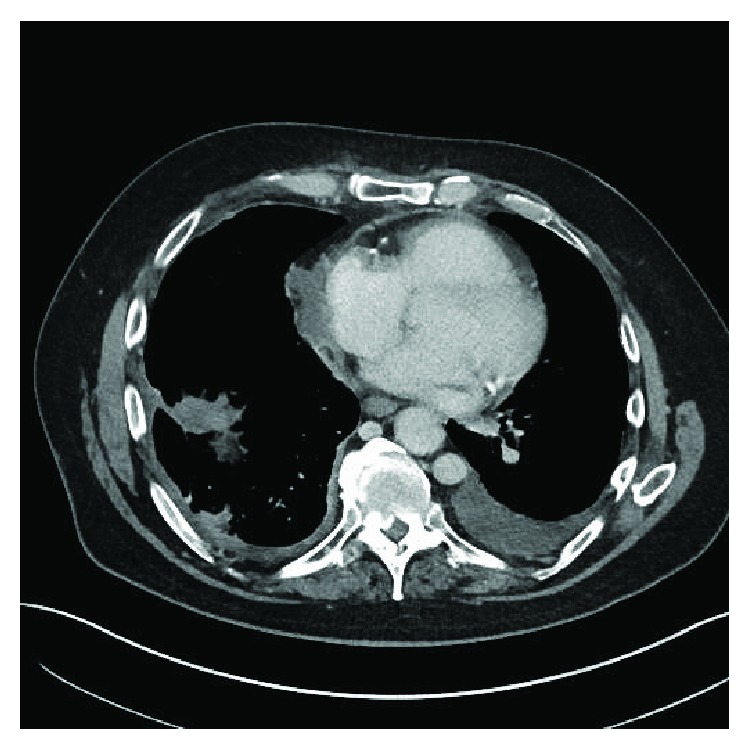
CT scan of the chest obtained one week after the pericardial window showing loculated pericardial fluid adjacent to the right atrium that is slightly higher in attenuation, suggestive of bloody or proteinaceous effusion.

One week after the pericardial window, the patient underwent a computerized tomography (CT) scan of the chest, abdomen, and pelvis which did not show evidence of metastatic disease elsewhere. Given the recurrence of UC with metastasis to the pericardium, he was started on atezolizumab. Subsequently, he was noted to have recurrent pericardial effusion suggesting failure of treatment (see Figure 4). He was then started on pemetrexed, a folate antimetabolite chemotherapy drug, for metastatic UC with plans to complete 6 cycles.

## 3. Discussion

Metastases to the heart typically occur late in the course of malignant disease. The incidence of cardiac metastasis, regardless of primary, has been estimated from 2.3% to 18.3% [5]. The pericardium is involved in about two thirds of all cardiac metastasis. Bussani et al. reported a large autopsy case series of 18,751 in-hospital deceased patients and found one or more malignant neoplasms in 7289 patients. The incidence of cardiac metastasis diagnosed postmortem was 9.1% and the tumors leading to the highest rates of cardiac metastasis were pleural mesothelioma (48.4%), melanoma (27.8%), lung adenocarcinoma (21%), undifferentiated carcinomas (19.5%), lung squamous cell carcinoma (18.2%), and breast carcinoma (15.5%). In contrast, only 3.9% of autopsied cases of UC revealed cardiac metastases.

To the best of our knowledge after a review of the literature, there have been only five cases of cardiac tamponade due to metastatic UC, with our case being the sixth [6–10]. In four of these cases, patients presented with symptoms of dyspnea, cough, fatigue, orthopnea, and chest pain [7–10]. In all cases, a chest radiograph showed the enlargement of the cardiac silhouette. An echocardiogram was utilized to evaluate the extent of pericardial effusion. Pericardiocentesis was performed for drainage and sent for cytology in all cases. However, in three of the cases, the pericardial fluid cytodiagnosis was equivocal and a pericardial biopsy was also obtained which led to the diagnosis of metastasis from UC [6, 8, 10]. In one case, the cytology was negative and no pericardial biopsy was performed; however, a large echogenic mass abutting the proximal free wall of the left ventricle and extending into the base of the pericardial cavity was noted [9]. In our case, pursuing a surgical approach for the pericardial window allowed us to obtain a pericardial biopsy in addition to pericardial fluid analysis in a patient with a prior history of malignancy. Both the pericardial fluid and pericardial biopsy showed malignant cells, which helped us establish the diagnosis of metastasis UC and guide appropriate management for the patient.

Of note, a minimum of 60 ml of pericardial fluid is necessary to ensure that benign diagnosis is truly benign and improves the likelihood of detecting malignancy with a sensitivity of 91.7%–92.1% [11, 12]. The false negative rates of cytology and biopsy range from 4% to 14.7% and 40% to 54.5%, respectively [13–15]. However, combining pericardial biopsy with pericardial effusion cytology further increases overall sensitivity for identifying malignancy by approximately 8% [9]. In this case, performing the surgical approach for the window allowed us to not only obtain a pericardial biopsy but also decompress the pericardial effusion and provide immediate restoration of improved hemodynamics and help prevent immediate recurrence of the pericardial effusion. Therefore, in patients with a history of malignancy, this approach should be considered.

Systemic chemotherapy is the standard approach for the initial treatment of patients with metastatic UC. However, the optimum management of UC with cardiac metastasis remains unclear at this time. In case of symptomatic cardiac metastases, the prognosis is extremely poor. Hattori et al., in their review of 14 reported cases in the literature found that most patients died within one year after diagnosis of symptomatic cardiac metastases from UC [7].

In patients with a history of malignancy who present with clinical, ECG, or CXR findings concerning pericardial effusion, one must consider a diagnosis of cardiac tamponade, particularly in a hemodynamically unstable patient. Cardiac metastasis should be on the differential diagnosis as an etiology. An echocardiogram may be performed for confirmation. However, if the patient is hemodynamically unstable, we recommend emergent pericardiocentesis, preferably with pericardial biopsy via surgical approach for accurate and early diagnosis of cardiac metastasis and for rapid relief of hemodynamic compromise. Treatment options for metastatic UC include systemic chemotherapy; however, prognosis of symptomatic cardiac metastasis from UC remains extremely poor.
