# Corrigendum to “A Rare Presentation of Cardiac Tamponade from Metastatic Urothelial Carcinoma of the Bladder”

**DOI:** 10.1155/2019/8376360

**Published:** 2019-09-12

**Authors:** Sowmya Palam, Ridhima Kapoor, Amrou Abdelkader, Jacquelyn Kulinski

**Affiliations:** ^1^Department of Internal Medicine, Medical College of Wisconsin, 9200 W. Wisconsin Ave., Suite 5100, Milwaukee, WI 53226, USA; ^2^Medical College of Wisconsin Department of Pathology, USA

In the article titled “A Rare Presentation of Cardiac Tamponade from Metastatic Urothelial Carcinoma of the Bladder” [1], Dr. Amrou Abdelkader was missing from the authors' list. Dr. Amrou Abdelkader was involved in writing and editing the pathology portion of the article and providing the pathology photos (Figures 3(a)–3(d)) and the associated legend. The corrected authors' list is shown above.
